# Synthesis and crystal structure of 3-(2-{3-[2-(2-oxooxazolidin-3-yl)eth­oxy]quinoxalin-2-yl­oxy}eth­yl)oxazolidin-2-one

**DOI:** 10.1107/S2056989025003755

**Published:** 2025-05-02

**Authors:** Fatima Ezzahra Aboutofil, Nour El Hoda Mustaphi, Olivier Blacque, Tuncer Hökelek, Ahmed Mazzah, Lhoussaine El Ghayati, Nada Kheira Sebbar

**Affiliations:** a Laboratory of Heterocyclic Organic Chemistry Medicines Science Research Center Pharmacochemistry Competence Center Mohammed V University in Rabat Faculté des Sciences Av Ibn Battouta BP 1014 Rabat Morocco; b University of Zurich, Department of Chemistry, Winterthurerstrasse 190, CH-8057 Zurich, Switzerland; c Department of Physics, Hacettepe University, 06800 Beytepe, Ankara, Türkiye; d Science and Technology of Lille USR 3290, Villeneuve d’ascq cedex, France; e Laboratory of Organic and Physical Chemistry, Applied Bioorganic Chemistry Team, Faculty of Sciences, Ibnou Zohr University, Agadir, Morocco; f Laboratory of Plant Chemistry Organic and Bioorganic Synthesis Faculty of Sciences Mohammed V University in Rabat 4 Avenue Ibn Battouta BP 1014 RP Rabat Morocco; University of Aberdeen, United Kingdom

**Keywords:** crystal structure, hydrogen bond, π-stacking, quinoxaline

## Abstract

In the title compound, C_18_H_20_N_4_O_6_, one of the oxazolidine rings adopts a twisted conformation and the other is a shallow envelope. In the crystal, weak C—H⋯O hydrogen bonds and π–π stacking inter­actions help to consolidate a three-dimensional architecture. The Hirshfeld surface analysis of the crystal structure indicates that the most important contributions for the crystal packing are from H⋯H (48.4%) and H⋯O/O⋯H (29.1%) contacts.

## Abstract 2

In the title compound, one of the oxazolidine rings adopts a twisted conformation and the other is a shallow envelope. In the crystal, weak C—H⋯O hydrogen bonds and π–π stacking inter­actions help to consolidate a three-dimensional architecture.

## 1. Chemical context

Quinoxaline and its derivatives are widely used in various fields, including medicine (Kaushal *et al.*, 2019 ▸; Montana *et al.*, 2019 ▸), pharmacology, mol­ecular biology, neuroscience, immunology, microbiology, agriculture, chemistry, toxicology, materials science, and biochemistry (Balderas-Renteria *et al.*, 2012 ▸; Pereira *et al.*, 2015 ▸; Zeb *et al.*, 2014 ▸; Tangherlini *et al.*, 2019 ▸; Vieira *et al.*, 2014 ▸; Zheng *et al.*, 2002 ▸).

The quinoxaline mol­ecule has been utilized as a precursor for synthesizing bioactive derivatives, with several research teams emphasizing its potential applications in the pharmaceutical and therapeutic fields (Raoa *et al.*, 2010 ▸; Yousra *et al.*, 2023 ▸). Different synthesis methodologies have been detailed in the literature, reflecting extensive research efforts to elucidate these compounds’ properties and applications (*e.g*., Gu *et al.*, 2017 ▸). Building on our previous research into the synthesis of quinoxaline derivatives (Yousra *et al.*, 2023 ▸), we have synthesized the title compound, C_18_H_20_N_4_O_6_ (**I**), and we now describe its synthesis, crystal structure and Hirshfeld surface.
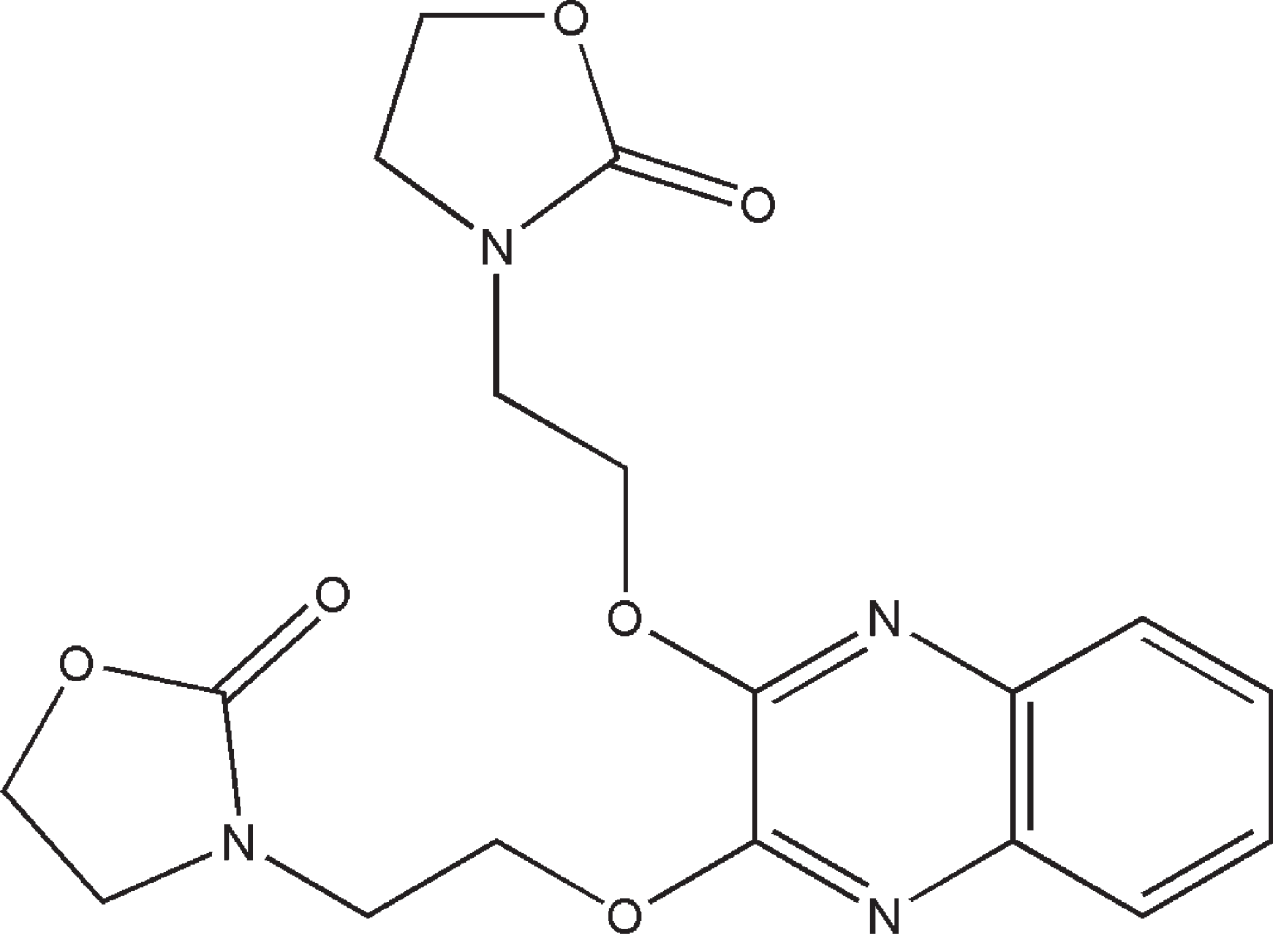


## 2. Structural commentary

**Figure 1:**
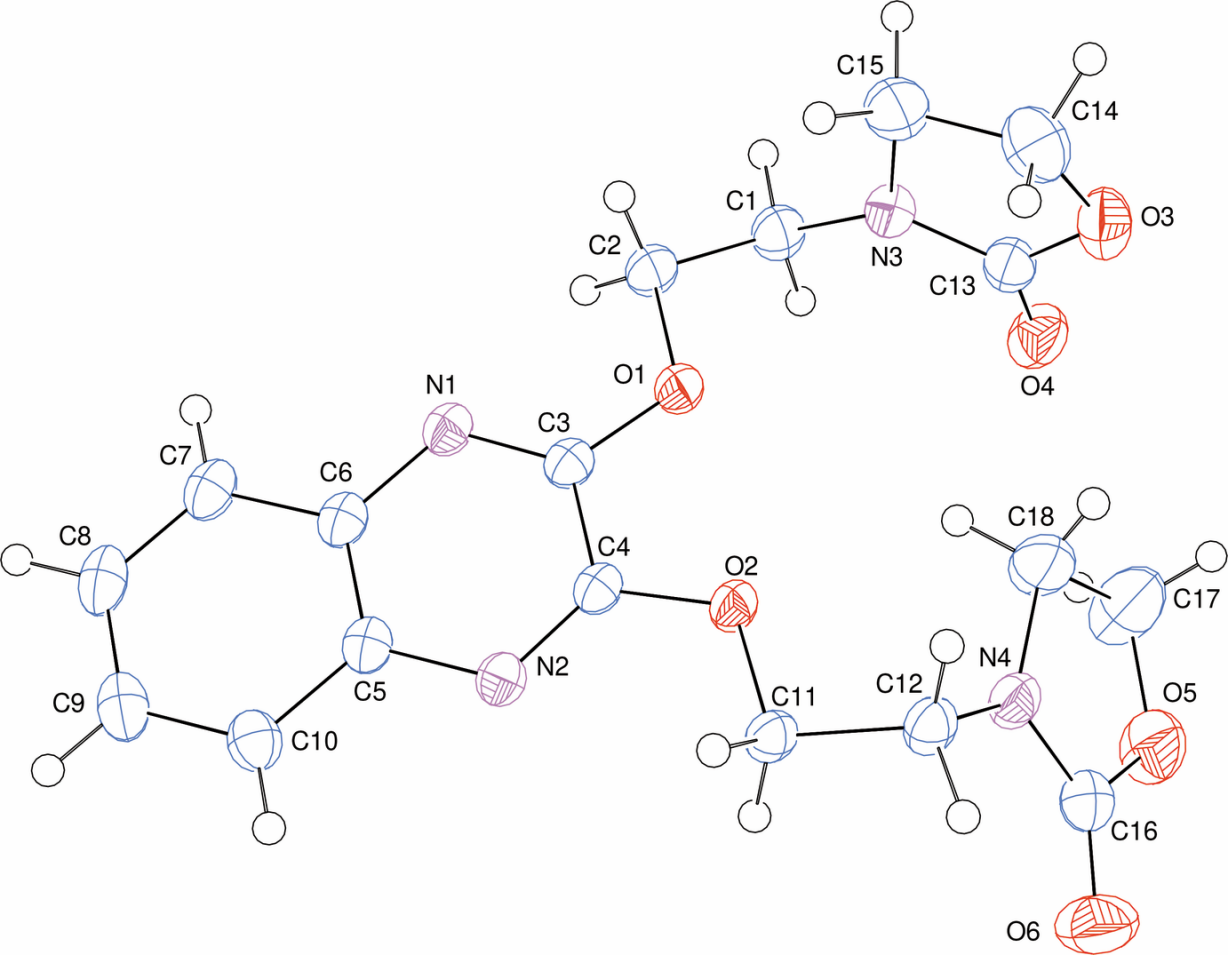
The mol­ecular structure of the title mol­ecule with 50% probability ellipsoids.

Compound (**I**) contains an almost planar quinoxaline fused ring and two oxazolidine rings (Fig. 1 ▸), where the oxazolidine (*C*, N3/O3/C13–C15) and (*D*, N4/O5/C16–C18) rings are in half-chair [with a puckering parameter value of φ = 305.0 (4)°] and shallow envelope conformations, respectively. In ring *D*, atom N4 is at the flap position and is 0.0849 (11) Å away from the best least-squares plane of the other four atoms. The almost planar *A* (N1/N2/C3–C6) and *B* (C5–C10) rings are oriented at a dihedral angle of 1.46 (4)°. Atoms O1, O2 and C11 are −0.094 (1), 0.059 (1) and 0.070 (1) Å, respectively, away from the best least-squares plane of ring *A*. The side chains both have *anti*–*gauche* conformations as indicated by the following torsion angles: C3—O1—C2—C1 = −162.00 (10), O1—C2—C1—N3 = −55.36 (14), C4—O2—C11—C12 = −174.35 (9) and O2—C11—C12—N4 = −57.76 (13)°. The dihedral angles between the quinoxaline ring and the pendant oxazolidine *C* and *D* rings (all atoms) are 85.72 (6) and 56.91 (7)°, respectively; the equivalent angle between the oxazolidine rings is 89.98 (9)°.

## 3. Supra­molecular features

**Figure 2:**
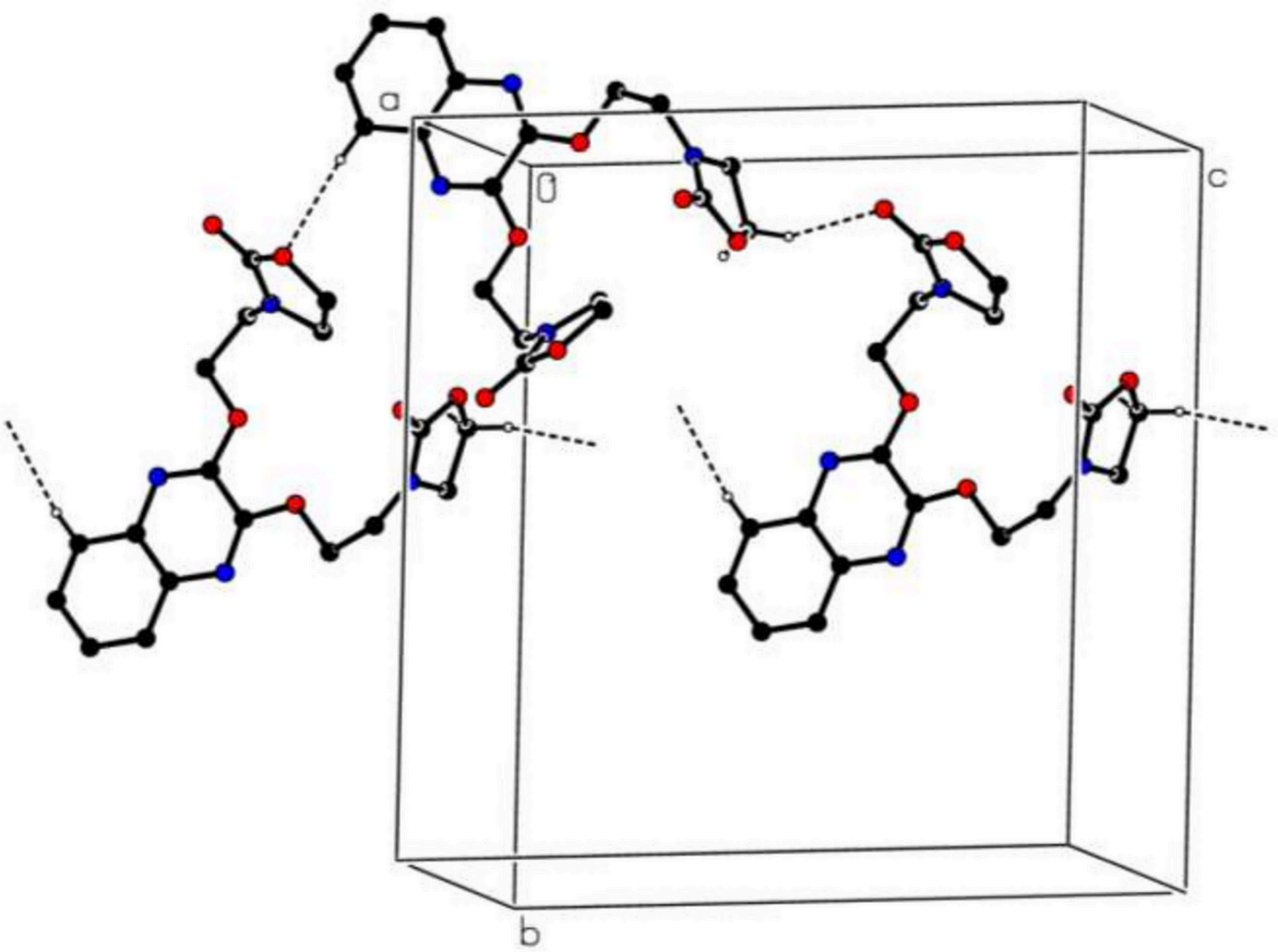
A partial packing diagram viewed down the *a*-axis direction. Inter­molecular C—H⋯O hydrogen bonds are shown as dashed lines. H atoms not involved in these inter­actions are omitted for clarity.

In the crystal structure of (**I**), the molecules are linked by C—H⋯O hydrogen bonds (Table 1 ▸ and Fig. 2 ▸). Aromatic π–π stacking inter­actions between the quinoxaline *A* and *B* rings of adjacent mol­ecules with a shortest inter­centroid distance of 3.5155 (7) Å may help to consolidate the packing. No C—H⋯π inter­actions could be identified.

## 4. Hirshfeld surface analysis

**Figure 3:**
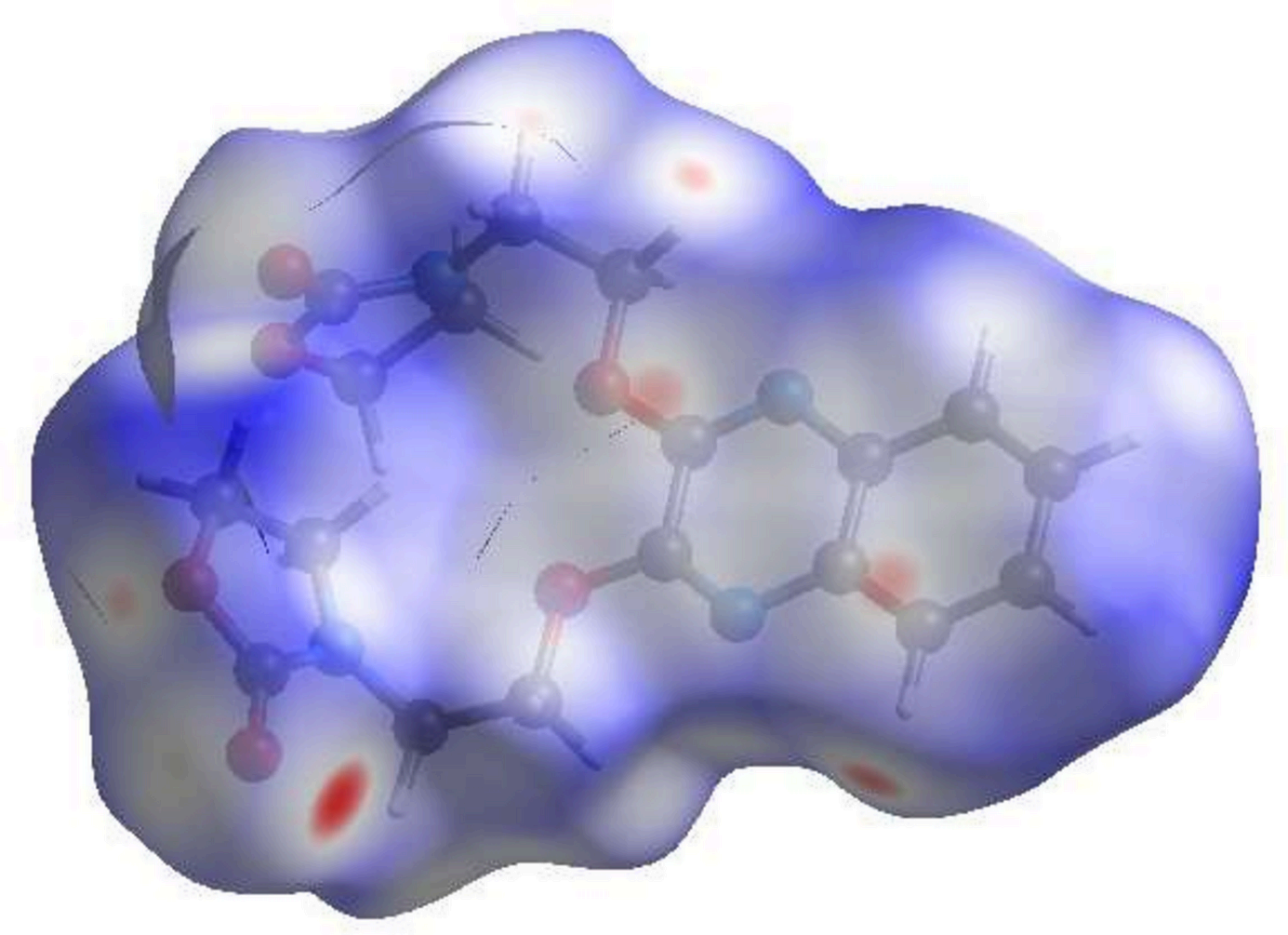
View of the three-dimensional Hirshfeld surface of the title compound plotted over *d*_norm_.

**Figure 4:**
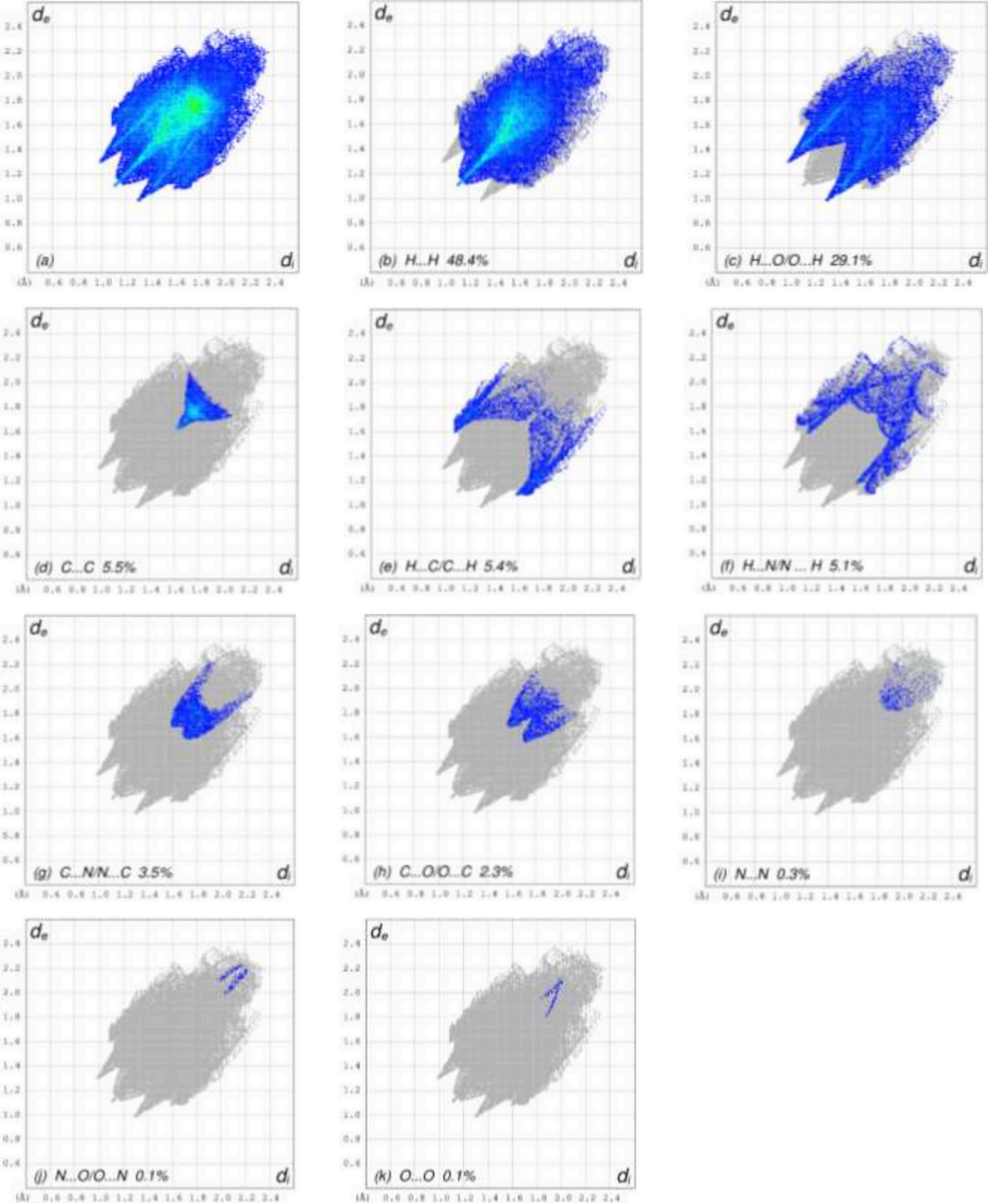
The full two-dimensional fingerprint plots for the title compound, showing (*a*) all inter­actions, and delineated into (*b*) H⋯H, (*c*) H⋯O/O⋯H, (*d*) C⋯C, (*e*) H⋯C/C⋯H, (*f*) H⋯N/N⋯H, (*g*) C⋯N/N⋯C, (*h*) C⋯O/O⋯C, (i) N⋯N, (*j*) N⋯O/O⋯N and (*k*) O⋯O inter­actions. The *d*_i_ and *d*_e_ values are the closest inter­nal and external distances (in Å) from given points on the Hirshfeld surface.

A Hirshfeld surface (HS) analysis was carried out using *Crystal Explorer 17.5* (Spackman *et al.*, 2021 ▸) to investigate the inter­molecular inter­actions in the crystal of (**I**). The HS is shown in Fig. 3 ▸, where the bright-red spots correspond to the respective donors and/or acceptors. According to the two-dimensional fingerprint plots (McKinnon *et al.*, 2007 ▸), the inter­molecular H⋯H and H⋯O/O⋯H contacts make the most important contributions to the HS of 48.4% and 29.1%, respectively (Fig. 4 ▸). All other contact types contribute 5% or less to the surface.

## 5. Database survey

A search of the Cambridge Structural Database (CSD) (Groom *et al.*, 2016 ▸; updated to January 2024) using the search fragment (**II**) yielded 25 hits of which those most similar to the title mol­ecule have the formula (**III**) with *R* = Me and *R*′ = CH_2_CO_2_H (CSD refcode DEZJAW; Missioui *et al.*, 2018 ▸) or benzyl (DUSHUV; Ramli *et al.*, 2010 ▸) with *R* = CF_3_ and *R*′ = *i*-Bu (DUBPUO; Wei *et al.*, 2019 ▸), with *R* = Ph and *R*′ = CH_2_ (cyclo-CHCH_2_O) and *R*′ = benzyl (PUGGII; Benzeid *et al.*, 2009 ▸). As expected, in all these hits, the di­hydro­quinoxaline ring system is essentially planar with the dihedral angle between the constituent rings being less than 1° or having the nitro­gen atom bearing the exocyclic substituent less than 0.03 Å from the mean plane of the remaining nine atoms.
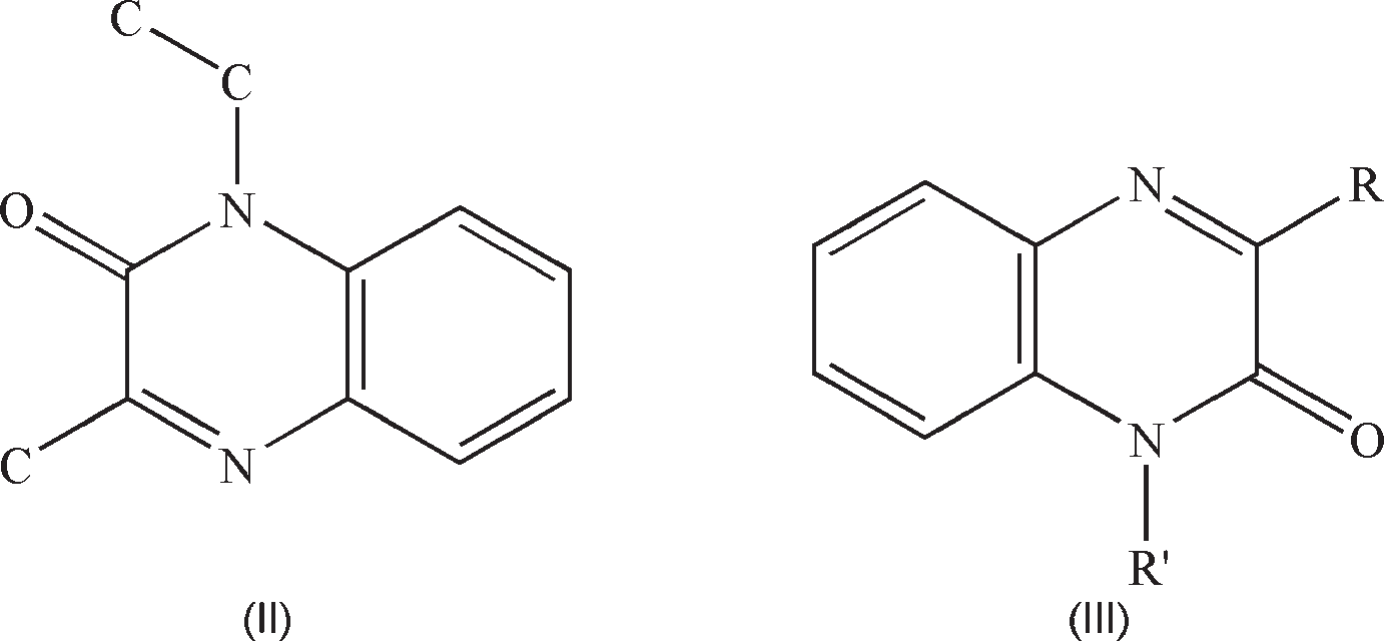


## 6. Synthesis and crystallization

A solution of quinoxaline-2,3-dione (0.29 g, 1.00 mmol) in di­methyl­formamide (15 ml) was prepared. To this solution, tetra-*n*-butyl­ammonium bromide (0.1 mmol), 2.2 equivalents of bis­(2-chloro­eth­yl)amine hydro­chloride, and 2.00 equivalents of potassium carbonate were added. The mixture was stirred at 353 K for 6 h. After stirring, the salts were removed by filtration, and the solution was evaporated under reduced pressure. The resulting residue was dissolved in di­chloro­methane. The remaining salts were extracted with distilled water. The mixture obtained was then chromatographed on a silica gel column using an eluent of ethyl acetate and hexane in a 4:1 ratio. The solid isolate was recrystallized from an ethanol solution, resulting in crystals of (**I**) with a yield of 56%.

## 7. Refinement

Crystal data, data collection and structure refinement details are summarized in Table 2 ▸. The C-bound hydrogen-atom positions were calculated geometrically at distances of 0.95 Å (for aromatic CH) and 0.99 Å (for CH_2_) and they were refined using a riding model by applying the constraint *U*_iso_(H) = 1.2*U*_eq_(C).

## Supplementary Material

Crystal structure: contains datablock(s) I. DOI: 10.1107/S2056989025003755/hb8134sup1.cif

Structure factors: contains datablock(s) I. DOI: 10.1107/S2056989025003755/hb8134Isup2.hkl

Supporting information file. DOI: 10.1107/S2056989025003755/hb8134Isup3.cdx

Supporting information file. DOI: 10.1107/S2056989025003755/hb8134Isup4.cml

CCDC reference: 2447382

Additional supporting information:  crystallographic information; 3D view; checkCIF report

## Figures and Tables

**Table 1:** Hydrogen-bond geometry (Å, °)

*D*—H⋯*A*	*D*—H	H⋯*A*	*D*⋯*A*	*D*—H⋯*A*
C14—H14*A*⋯O6^i^	0.99	2.37	3.189 (2)	140
C10—H10⋯O5^ii^	0.95	2.56	3.4935 (18)	168

Symmetry codes: (i) 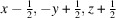; (ii) 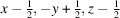.

**Table 2:** Experimental details

Crystal data	
Chemical formula	C_18_H_20_N_4_O_6_
*M*_r_	388.38
Crystal system, space group	Monoclinic, *P*2_1_/*n*
Temperature (K)	160
*a*, *b*, *c* (Å)	6.6576 (1), 17.1463 (2), 15.8105 (2)
β (°)	98.935 (1)
*V* (Å^3^)	1782.92 (4)
*Z*	4
Radiation type	Cu *K*α
μ (mm^−1^)	0.93
Crystal size (mm)	0.25 × 0.21 × 0.10

Data collection	
Diffractometer	XtaLAB Synergy, Dualflex, HyPix
Absorption correction	Analytical (*CrysAlis PRO*; Rigaku OD, 2024 ▸)
*T*_min_, *T*_max_	0.845, 0.932
No. of measured, independent and observed [*I* > 2σ(*I*)] reflections	23056, 3781, 3577
*R*_int_	0.027
(sin θ/λ)_max_ (Å^−1^)	0.633

Refinement	
*R*[*F*^2^ > 2σ(*F*^2^)], *wR*(*F*^2^), *S*	0.038, 0.099, 1.06
No. of reflections	3781
No. of parameters	254
H-atom treatment	H-atom parameters constrained
Δρ_max_, Δρ_min_ (e Å^−3^)	0.35, −0.29

Computer programs: *CrysAlis PRO* (Rigaku OD, 2024 ▸), *SHELXT* (Sheldrick, 2015*a* ▸), *SHELXL2019/3* (Sheldrick, 2015*b* ▸) and *OLEX2* (Dolomanov *et al.*, 2009 ▸).

## supplementary crystallographic information

### 3-(2-{3-[2-(2-Oxooxazolidin-3-yl)ethoxy]quinoxalin-2-yloxy}ethyl)oxazolidin-2-one Crystal data

**Table:**

C_18_H_20_N_4_O_6_	*F*(000) = 816
*M**_r_* = 388.38	*D*_x_ = 1.447 Mg m^−^^3^
Monoclinic, *P*2_1_/*n*	Cu *K*α radiation, λ = 1.54184 Å
*a* = 6.6576 (1) Å	Cell parameters from 17516 reflections
*b* = 17.1463 (2) Å	θ = 3.8–79.5°
*c* = 15.8105 (2) Å	µ = 0.93 mm^−^^1^
β = 98.935 (1)°	*T* = 160 K
*V* = 1782.92 (4) Å^3^	Plate, colourless
*Z* = 4	0.25 × 0.21 × 0.10 mm

### 3-(2-{3-[2-(2-Oxooxazolidin-3-yl)ethoxy]quinoxalin-2-yloxy}ethyl)oxazolidin-2-one Data collection

**Table:**

XtaLAB Synergy, Dualflex, HyPix diffractometer	3781 independent reflections
Radiation source: micro-focus sealed X-ray tube, PhotonJet (Cu) X-ray Source	3577 reflections with *I* > 2σ(*I*)
Mirror monochromator	*R*_int_ = 0.027
Detector resolution: 10.0000 pixels mm^-1^	θ_max_ = 77.4°, θ_min_ = 3.8°
ω scans	*h* = −8→8
Absorption correction: analytical (CrysAlisPro; Rigaku OD, 2024)	*k* = −21→21
*T*_min_ = 0.845, *T*_max_ = 0.932	*l* = −17→19
23056 measured reflections	

### 3-(2-{3-[2-(2-Oxooxazolidin-3-yl)ethoxy]quinoxalin-2-yloxy}ethyl)oxazolidin-2-one Refinement

**Table:**

Refinement on *F*^2^	Hydrogen site location: inferred from neighbouring sites
Least-squares matrix: full	H-atom parameters constrained
*R*[*F*^2^ > 2σ(*F*^2^)] = 0.038	*w* = 1/[σ^2^(*F*_o_^2^) + (0.0447*P*)^2^ + 0.6483*P*] where *P* = (*F*_o_^2^ + 2*F*_c_^2^)/3
*wR*(*F*^2^) = 0.099	(Δ/σ)_max_ < 0.001
*S* = 1.06	Δρ_max_ = 0.35 e Å^−^^3^
3781 reflections	Δρ_min_ = −0.29 e Å^−^^3^
254 parameters	Extinction correction: *SHELXL2019/3* (Sheldrick, 2015b), Fc^*^=kFc[1+0.001xFc^2^λ^3^/sin(2θ)]^-1/4^
0 restraints	Extinction coefficient: 0.0023 (2)
Primary atom site location: dual	

### 3-(2-{3-[2-(2-Oxooxazolidin-3-yl)ethoxy]quinoxalin-2-yloxy}ethyl)oxazolidin-2-one Special details

**Table:**

Geometry. All esds (except the esd in the dihedral angle between two l.s. planes) are estimated using the full covariance matrix. The cell esds are taken into account individually in the estimation of esds in distances, angles and torsion angles; correlations between esds in cell parameters are only used when they are defined by crystal symmetry. An approximate (isotropic) treatment of cell esds is used for estimating esds involving l.s. planes.

### 3-(2-{3-[2-(2-Oxooxazolidin-3-yl)ethoxy]quinoxalin-2-yloxy}ethyl)oxazolidin-2-one Fractional atomic coordinates and isotropic or equivalent isotropic displacement parameters (Å^2^)

**Table:**

	*x*	*y*	*z*	*U*_iso_*/*U*_eq_	
O1	0.34531 (13)	0.47199 (5)	0.72202 (5)	0.02841 (19)	
O2	0.29395 (12)	0.35049 (4)	0.62370 (5)	0.02608 (19)	
O3	0.35795 (16)	0.33324 (6)	0.96229 (7)	0.0444 (3)	
O4	0.66392 (15)	0.37070 (6)	0.93404 (7)	0.0451 (3)	
O5	0.74284 (18)	0.16514 (7)	0.76528 (7)	0.0527 (3)	
O6	0.62002 (19)	0.11202 (8)	0.63787 (7)	0.0587 (3)	
N1	0.28423 (15)	0.55758 (6)	0.60819 (6)	0.0269 (2)	
N2	0.25336 (14)	0.42434 (6)	0.50000 (6)	0.0253 (2)	
N3	0.37902 (16)	0.44689 (6)	0.89855 (7)	0.0322 (2)	
N4	0.44993 (17)	0.20867 (6)	0.69704 (7)	0.0331 (2)	
C1	0.4760 (2)	0.51244 (7)	0.86354 (8)	0.0342 (3)	
H1A	0.616539	0.497667	0.857060	0.041*	
H1B	0.484189	0.556502	0.904393	0.041*	
C2	0.3645 (2)	0.53887 (7)	0.77796 (8)	0.0328 (3)	
H2A	0.228380	0.559210	0.784233	0.039*	
H2B	0.441442	0.580793	0.754216	0.039*	
C3	0.30273 (16)	0.48736 (7)	0.63769 (7)	0.0252 (2)	
C4	0.28227 (16)	0.41917 (6)	0.58255 (7)	0.0241 (2)	
C5	0.23938 (16)	0.49903 (7)	0.46633 (8)	0.0261 (2)	
C6	0.25158 (16)	0.56504 (7)	0.51985 (8)	0.0266 (2)	
C7	0.23349 (18)	0.64004 (7)	0.48354 (9)	0.0316 (3)	
H7	0.240283	0.684707	0.519426	0.038*	
C8	0.20594 (18)	0.64869 (8)	0.39597 (9)	0.0354 (3)	
H8	0.193559	0.699445	0.371627	0.043*	
C9	0.19602 (19)	0.58335 (8)	0.34250 (9)	0.0358 (3)	
H9	0.178341	0.590080	0.282161	0.043*	
C10	0.21172 (18)	0.50923 (8)	0.37678 (8)	0.0314 (3)	
H10	0.203901	0.465090	0.340091	0.038*	
C11	0.27737 (18)	0.28136 (6)	0.57133 (7)	0.0266 (2)	
H11A	0.395000	0.277258	0.540209	0.032*	
H11B	0.151217	0.282899	0.528962	0.032*	
C12	0.27329 (19)	0.21297 (7)	0.63101 (8)	0.0298 (3)	
H12A	0.149795	0.216567	0.658525	0.036*	
H12B	0.263947	0.164211	0.597081	0.036*	
C13	0.4844 (2)	0.38405 (7)	0.93076 (8)	0.0335 (3)	
C14	0.1517 (2)	0.36156 (10)	0.94072 (11)	0.0483 (4)	
H14A	0.077858	0.356549	0.990193	0.058*	
H14B	0.077252	0.332043	0.891836	0.058*	
C15	0.1730 (2)	0.44643 (9)	0.91721 (10)	0.0429 (3)	
H15A	0.073565	0.461243	0.866518	0.051*	
H15B	0.157556	0.481479	0.965547	0.051*	
C16	0.6022 (2)	0.15857 (8)	0.69375 (8)	0.0366 (3)	
C17	0.6778 (4)	0.22219 (12)	0.82074 (12)	0.0720 (6)	
H17A	0.773792	0.266660	0.828125	0.086*	
H17B	0.670734	0.199276	0.877681	0.086*	
C18	0.4690 (3)	0.24899 (9)	0.77867 (9)	0.0520 (4)	
H18A	0.361999	0.232637	0.811984	0.062*	
H18B	0.463720	0.306297	0.771004	0.062*	

### 3-(2-{3-[2-(2-Oxooxazolidin-3-yl)ethoxy]quinoxalin-2-yloxy}ethyl)oxazolidin-2-one Atomic displacement parameters (Å^2^)

**Table:**

	*U*^11^	*U*^22^	*U*^33^	*U*^12^	*U*^13^	*U*^23^
O1	0.0383 (5)	0.0205 (4)	0.0264 (4)	0.0002 (3)	0.0047 (3)	−0.0015 (3)
O2	0.0325 (4)	0.0185 (4)	0.0268 (4)	0.0001 (3)	0.0032 (3)	0.0000 (3)
O3	0.0531 (6)	0.0357 (5)	0.0436 (5)	−0.0012 (4)	0.0054 (4)	0.0096 (4)
O4	0.0414 (6)	0.0437 (6)	0.0480 (6)	0.0111 (4)	−0.0001 (4)	0.0032 (5)
O5	0.0546 (7)	0.0533 (6)	0.0442 (6)	0.0125 (5)	−0.0111 (5)	0.0030 (5)
O6	0.0615 (7)	0.0665 (8)	0.0468 (6)	0.0281 (6)	0.0044 (5)	−0.0123 (6)
N1	0.0261 (5)	0.0223 (5)	0.0326 (5)	0.0002 (4)	0.0051 (4)	0.0015 (4)
N2	0.0236 (5)	0.0241 (5)	0.0280 (5)	0.0001 (3)	0.0028 (4)	0.0012 (4)
N3	0.0364 (6)	0.0299 (5)	0.0304 (5)	0.0043 (4)	0.0053 (4)	0.0020 (4)
N4	0.0451 (6)	0.0238 (5)	0.0285 (5)	0.0043 (4)	−0.0010 (4)	−0.0016 (4)
C1	0.0425 (7)	0.0263 (6)	0.0330 (6)	−0.0020 (5)	0.0034 (5)	−0.0029 (5)
C2	0.0464 (7)	0.0216 (5)	0.0300 (6)	0.0009 (5)	0.0051 (5)	−0.0039 (5)
C3	0.0233 (5)	0.0232 (5)	0.0295 (6)	0.0001 (4)	0.0054 (4)	0.0003 (4)
C4	0.0220 (5)	0.0207 (5)	0.0297 (6)	0.0002 (4)	0.0047 (4)	0.0011 (4)
C5	0.0202 (5)	0.0262 (6)	0.0318 (6)	−0.0001 (4)	0.0042 (4)	0.0044 (4)
C6	0.0204 (5)	0.0255 (6)	0.0342 (6)	0.0005 (4)	0.0053 (4)	0.0042 (4)
C7	0.0263 (6)	0.0252 (6)	0.0436 (7)	0.0005 (4)	0.0061 (5)	0.0059 (5)
C8	0.0275 (6)	0.0324 (6)	0.0464 (7)	0.0019 (5)	0.0057 (5)	0.0153 (5)
C9	0.0300 (6)	0.0417 (7)	0.0354 (6)	0.0012 (5)	0.0042 (5)	0.0130 (5)
C10	0.0281 (6)	0.0348 (6)	0.0310 (6)	−0.0006 (5)	0.0032 (5)	0.0040 (5)
C11	0.0305 (6)	0.0205 (5)	0.0280 (5)	−0.0003 (4)	0.0024 (4)	−0.0025 (4)
C12	0.0343 (6)	0.0217 (5)	0.0328 (6)	−0.0017 (4)	0.0032 (5)	−0.0002 (4)
C13	0.0436 (7)	0.0298 (6)	0.0257 (6)	0.0017 (5)	0.0007 (5)	−0.0019 (5)
C14	0.0453 (8)	0.0538 (9)	0.0464 (8)	−0.0081 (7)	0.0084 (6)	0.0063 (7)
C15	0.0377 (7)	0.0479 (8)	0.0438 (8)	0.0052 (6)	0.0090 (6)	0.0029 (6)
C16	0.0415 (7)	0.0344 (7)	0.0331 (6)	0.0043 (5)	0.0033 (5)	0.0041 (5)
C17	0.1003 (15)	0.0538 (10)	0.0487 (9)	0.0250 (10)	−0.0297 (10)	−0.0142 (8)
C18	0.0775 (11)	0.0436 (8)	0.0309 (7)	0.0155 (8)	−0.0045 (7)	−0.0085 (6)

### 3-(2-{3-[2-(2-Oxooxazolidin-3-yl)ethoxy]quinoxalin-2-yloxy}ethyl)oxazolidin-2-one Geometric parameters (Å, º)

**Table:**

O1—C2	1.4416 (14)	C5—C6	1.4079 (17)
O1—C3	1.3454 (14)	C5—C10	1.4100 (17)
O2—C4	1.3418 (13)	C6—C7	1.4057 (16)
O2—C11	1.4403 (13)	C7—H7	0.9500
O3—C13	1.3589 (17)	C7—C8	1.3762 (19)
O3—C14	1.447 (2)	C8—H8	0.9500
O4—C13	1.2101 (17)	C8—C9	1.399 (2)
O5—C16	1.3563 (17)	C9—H9	0.9500
O5—C17	1.425 (2)	C9—C10	1.3792 (18)
O6—C16	1.2100 (18)	C10—H10	0.9500
N1—C3	1.2901 (15)	C11—H11A	0.9900
N1—C6	1.3857 (16)	C11—H11B	0.9900
N2—C4	1.2924 (15)	C11—C12	1.5080 (16)
N2—C5	1.3846 (15)	C12—H12A	0.9900
N3—C1	1.4483 (17)	C12—H12B	0.9900
N3—C13	1.3421 (16)	C14—H14A	0.9900
N3—C15	1.4474 (18)	C14—H14B	0.9900
N4—C12	1.4480 (16)	C14—C15	1.514 (2)
N4—C16	1.3359 (17)	C15—H15A	0.9900
N4—C18	1.4522 (17)	C15—H15B	0.9900
C1—H1A	0.9900	C17—H17A	0.9900
C1—H1B	0.9900	C17—H17B	0.9900
C1—C2	1.5084 (18)	C17—C18	1.516 (3)
C2—H2A	0.9900	C18—H18A	0.9900
C2—H2B	0.9900	C18—H18B	0.9900
C3—C4	1.4521 (15)		
			
C3—O1—C2	115.92 (9)	C5—C10—H10	120.0
C4—O2—C11	116.75 (9)	C9—C10—C5	119.93 (12)
C13—O3—C14	108.53 (11)	C9—C10—H10	120.0
C16—O5—C17	109.50 (12)	O2—C11—H11A	110.4
C3—N1—C6	116.22 (10)	O2—C11—H11B	110.4
C4—N2—C5	116.25 (10)	O2—C11—C12	106.71 (9)
C13—N3—C1	122.03 (11)	H11A—C11—H11B	108.6
C13—N3—C15	111.99 (11)	C12—C11—H11A	110.4
C15—N3—C1	125.10 (11)	C12—C11—H11B	110.4
C12—N4—C18	124.44 (11)	N4—C12—C11	113.52 (10)
C16—N4—C12	122.74 (11)	N4—C12—H12A	108.9
C16—N4—C18	112.25 (11)	N4—C12—H12B	108.9
N3—C1—H1A	109.0	C11—C12—H12A	108.9
N3—C1—H1B	109.0	C11—C12—H12B	108.9
N3—C1—C2	112.93 (11)	H12A—C12—H12B	107.7
H1A—C1—H1B	107.8	O4—C13—O3	121.80 (12)
C2—C1—H1A	109.0	O4—C13—N3	128.51 (13)
C2—C1—H1B	109.0	N3—C13—O3	109.68 (12)
O1—C2—C1	107.19 (10)	O3—C14—H14A	110.7
O1—C2—H2A	110.3	O3—C14—H14B	110.7
O1—C2—H2B	110.3	O3—C14—C15	105.00 (12)
C1—C2—H2A	110.3	H14A—C14—H14B	108.8
C1—C2—H2B	110.3	C15—C14—H14A	110.7
H2A—C2—H2B	108.5	C15—C14—H14B	110.7
O1—C3—C4	115.02 (10)	N3—C15—C14	100.55 (11)
N1—C3—O1	122.32 (10)	N3—C15—H15A	111.7
N1—C3—C4	122.65 (11)	N3—C15—H15B	111.7
O2—C4—C3	115.00 (10)	C14—C15—H15A	111.7
N2—C4—O2	122.56 (10)	C14—C15—H15B	111.7
N2—C4—C3	122.44 (10)	H15A—C15—H15B	109.4
N2—C5—C6	121.22 (10)	O6—C16—O5	122.03 (13)
N2—C5—C10	119.43 (11)	O6—C16—N4	127.85 (13)
C6—C5—C10	119.35 (11)	N4—C16—O5	110.11 (12)
N1—C6—C5	121.12 (10)	O5—C17—H17A	110.4
N1—C6—C7	119.09 (11)	O5—C17—H17B	110.4
C7—C6—C5	119.78 (11)	O5—C17—C18	106.47 (13)
C6—C7—H7	120.0	H17A—C17—H17B	108.6
C8—C7—C6	119.93 (12)	C18—C17—H17A	110.4
C8—C7—H7	120.0	C18—C17—H17B	110.4
C7—C8—H8	119.7	N4—C18—C17	101.14 (13)
C7—C8—C9	120.54 (11)	N4—C18—H18A	111.5
C9—C8—H8	119.7	N4—C18—H18B	111.5
C8—C9—H9	119.8	C17—C18—H18A	111.5
C10—C9—C8	120.46 (12)	C17—C18—H18B	111.5
C10—C9—H9	119.8	H18A—C18—H18B	109.4
			
O1—C3—C4—O2	4.66 (14)	C6—C5—C10—C9	−0.28 (17)
O1—C3—C4—N2	−175.86 (10)	C6—C7—C8—C9	−0.10 (18)
O2—C11—C12—N4	−57.76 (13)	C7—C8—C9—C10	0.64 (19)
O3—C14—C15—N3	19.93 (15)	C8—C9—C10—C5	−0.44 (19)
O5—C17—C18—N4	−6.2 (2)	C10—C5—C6—N1	−178.23 (10)
N1—C3—C4—O2	−176.22 (10)	C10—C5—C6—C7	0.82 (16)
N1—C3—C4—N2	3.25 (18)	C11—O2—C4—N2	1.41 (15)
N1—C6—C7—C8	178.43 (10)	C11—O2—C4—C3	−179.12 (9)
N2—C5—C6—N1	1.99 (16)	C12—N4—C16—O5	−177.58 (11)
N2—C5—C6—C7	−178.96 (10)	C12—N4—C16—O6	1.4 (2)
N2—C5—C10—C9	179.50 (11)	C12—N4—C18—C17	178.95 (14)
N3—C1—C2—O1	−55.36 (14)	C13—O3—C14—C15	−17.07 (15)
C1—N3—C13—O3	177.45 (11)	C13—N3—C1—C2	132.78 (12)
C1—N3—C13—O4	−2.1 (2)	C13—N3—C15—C14	−17.38 (15)
C1—N3—C15—C14	173.26 (12)	C14—O3—C13—O4	−173.94 (13)
C2—O1—C3—N1	1.41 (16)	C14—O3—C13—N3	6.47 (15)
C2—O1—C3—C4	−179.46 (10)	C15—N3—C1—C2	−58.87 (16)
C3—O1—C2—C1	−162.00 (10)	C15—N3—C13—O3	7.71 (15)
C3—N1—C6—C5	0.10 (16)	C15—N3—C13—O4	−171.84 (14)
C3—N1—C6—C7	−178.95 (10)	C16—O5—C17—C18	3.4 (2)
C4—O2—C11—C12	−174.35 (9)	C16—N4—C12—C11	−102.91 (14)
C4—N2—C5—C6	−1.41 (16)	C16—N4—C18—C17	7.40 (18)
C4—N2—C5—C10	178.81 (10)	C17—O5—C16—O6	−177.74 (17)
C5—N2—C4—O2	178.41 (9)	C17—O5—C16—N4	1.34 (19)
C5—N2—C4—C3	−1.03 (16)	C18—N4—C12—C11	86.40 (16)
C5—C6—C7—C8	−0.63 (17)	C18—N4—C16—O5	−5.86 (17)
C6—N1—C3—O1	176.48 (9)	C18—N4—C16—O6	173.16 (16)
C6—N1—C3—C4	−2.57 (16)		

### 3-(2-{3-[2-(2-Oxooxazolidin-3-yl)ethoxy]quinoxalin-2-yloxy}ethyl)oxazolidin-2-one Hydrogen-bond geometry (Å, º)

**Table:**

*D*—H···*A*	*D*—H	H···*A*	*D*···*A*	*D*—H···*A*
C14—H14*A*···O6^i^	0.99	2.37	3.189 (2)	140
C10—H10···O5^ii^	0.95	2.56	3.4935 (18)	168

Symmetry codes: (i) *x*−1/2, −*y*+1/2, *z*+1/2; (ii) *x*−1/2, −*y*+1/2, *z*−1/2.
